# Improving T2D machine learning-based prediction accuracy with SNPs and younger age

**DOI:** 10.1016/j.csbj.2025.06.038

**Published:** 2025-06-23

**Authors:** Cynthia AL Hageh, Andreas Henschel, Hao Zhou, Jorge Zubelli, Moni Nader, Stephanie Chacar, Nantia Iakovidou, Haralampos Hatzikirou, Antoine Abchee, Siobhán O’Sullivan, Pierre A. Zalloua

**Affiliations:** a Department of Public Health & Epidemiology, Khalifa University, Abu Dhabi, United Arab Emirates; b Department of Computer Science, Khalifa University, Abu Dhabi, United Arab Emirates; c Department of Mathematics, Khalifa University, Abu Dhabi, United Arab Emirates; d ADIA Lab, Abu Dhabi, United Arab Emirates; e Department of Medical Sciences, Khalifa University, Abu Dhabi, United Arab Emirates; f Department of Informatics, Aristotle University of Thessaloniki, Greece; g Faculty of Medicine, University of Balamand, Lebanon; h Department of Biological Sciences, Khalifa University, Abu Dhabi, United Arab Emirates; i Harvard T.H. Chan School of Public Health, Boston, MA, USA; j Center for Cyber-Physical Systems, Khalifa University, Abu Dhabi, United Arab Emirates

**Keywords:** T2D, Machine Learning, AI, Predictive models

## Abstract

**Background:**

This study aimed to evaluate whether integrating clinical and genomic data improves the performance of machine learning (ML) models for predicting Type 2 Diabetes (T2D) risk.

**Methods:**

Six models—Random Forest, Support Vector Machine, Linear Discriminant Analysis, Logistic Regression, Gradient Boosting Machine, and Decision Tree—were trained and tested on a discovery dataset (N=3,546) and validated in the UK Biobank (N=31,620). Model performance was assessed using clinical data alone, combined clinical and genomic data, and in age-specific groups (>55 and ≤55 years).

**Results:**

The inclusion of genomic data modestly improved model performance across all algorithms in the discovery dataset. Clinical features such as family history of T2D and hypertension consistently ranked as top features. When SNPs were added, T2D-associated variants, including rs2943641 (*IRS1*), rs7903146 (*TCF7L2*), and rs7756992 (*CDKAL1*), emerged among the most important features, particularly in younger individuals. These findings demonstrate the translational potential of incorporating genomics for early risk identification. In the UK Biobank, all models achieved AUCs exceeding 91 % with combined clinical and genomic data. Performance was notably better among younger individuals (≤55 years), emphasizing the models’ potential for early detection. Integration of a polygenic risk score (PRS) further supported risk prediction, particularly in younger individuals, though incremental gains were modest.

**Conclusions:**

While traditional clinical factors remained the strongest predictors of T2D risk, integration of genomic data produced a modest improvement in model performance, especially among younger adults. Validation across independent datasets confirmed the generalizability of these findings, underscoring the value of multi-dimensional risk-prediction models to refine T2D risk assessment.

## Graphical Abstract

**Figure:**
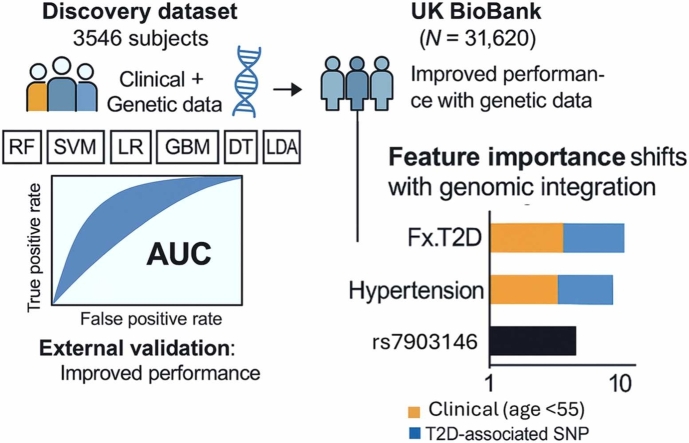

## 1 Introduction

Type 2 Diabetes (T2D) is a chronic metabolic disorder characterized by abnormal blood glucose levels due to ineffective insulin utilization or production. T2D accounts for most diabetes cases and poses a significant global health challenge due to its sudden onset and potential for severe complications. Nearly a third of patients with T2D are unaware of their condition, often due to asymptomatic presentation in early disease or prediabetes stages [1]. Undiagnosed or late-diagnosed T2D can lead to serious complications, including cardiovascular disease, kidney failure, and neuropathy, highlighting the urgent need for early detection and intervention [2].

Machine learning (ML) has emerged as a promising tool for disease prediction, offering the ability to process complex datasets and uncover patterns that conventional statistical methods might overlook [3]. ML algorithms are particularly effective, offering robust analysis capabilities without requiring high computational power [4]. Although ML algorithms are inherently suited to linear relationships, they can be adapted to model nonlinear relationships using various kernel methods. This adaptability makes them highly applicable for T2D risk evaluation and associated comorbidities, including cardiovascular diseases [4], [5]. Significantly, ML methods facilitate prospective risk prediction, identifying high-risk individuals before the onset of clinical symptoms [6]. Despite these advantages, significant challenges remain in achieving consistency and generalizability across ML-based T2D prediction models, particularly regarding feature selection and data integration [7].

Many studies investigating the use of predictive models for T2D prioritize prediction accuracy at the expense of model interpretability and generalizability by using more T2D impactful features like HbA1c or fasting blood sugar values while excluding potentially informative features. Some models rely on fewer than 20 features [8], and there is no standardized consensus on which features to include in T2D prediction models. Numerous data-driven models for T2D detection have been proposed [9], [10], [11]. Yet, most of these studies rely on laboratory-based biomarkers, such as fasting glucose and HbA1c, which are already established diagnostic markers for the disease [12], [13]. While these models show promising accuracy, their clinical utility is limited due to redundancy with conventional diagnostic tests [14]. For early disease identification and prevention especially in asymptomatic or resource-limited populations, greater emphasis should be placed on non-invasive predictors. These include sociodemographic characteristics, self-reported clinical history, and lifestyle-related [15], [16] factors that offer cost effective alternatives to laboratory based diagnostics [17]. Several studies have explored the development of such models using demographic and clinical variables to enhance accessibility and support real-world applications. In parallel, the integration of genomic data, particularly single-nucleotide polymorphisms (SNPs), offers additional value by capturing individual genetic predisposition to T2D. When combined with conventional non-invasive predictors, genomic features can improve model precision, enrich the predictive landscape and support the development of personalized prevention strategies.

ML has shown considerable promise in T2D prediction, with various methodologies such as classification, regression, and clustering tools offering distinct impacts depending on the specific clinical question being addressed [3]. Classification algorithms, including Support Vector Machine, Logistic Regression, and Decision Trees, are commonly used in medical domains, particularly for diagnosing diseases like T2D. Comparative studies have extensively benchmarked these models across performance metrics [18], [19] such as accuracy, precision, F1-score, and training time [20]. Such evaluations highlight the importance of supervised learning approaches, a key aspect of machine learning that utilizes labeled datasets to train models for accurate outcome prediction. Supervised learning includes two subclasses: classification, for categorical outputs, and regression, for continuous output ranges. Classification involves training a model on existing data to predict future behavior, with tasks including binary (two-class labels) and multi-class (multiple-class labels) classification [20]. ML approaches have been widely explored to improve the prediction of undiagnosed diabetes, achieving promising results in early detection strategies [17], [21], [22]. When integrated with genomic data, these models outperform traditional screening tools for stratifying individuals by risk [17], [21], [22].

This study evaluates the predictive performance of six ML-based models —Random Forest (RF), Support Vector Machine (SVM), Logistic Regression (LR), Gradient Boosting Machine (GBM), Linear Discriminant Analysis (LDA), and Decision Tree (DT) across two distinct input approaches: [1] clinical and epidemiologic data alone and [2] clinical, epidemiologic and genomic data (SNPs). Models were trained and internally validated in a discovery dataset (FGENTCARD, N = 3,546, non-European population), and externally replicated in the UK Biobank (N = 31,620, European population), to assess model generalizability. This study evaluated accuracy, precision, recall, and F-score to quantify the incremental value of genomic integration. Particular attention is given to younger adults, where early identification and preventative intervention may have the greatest impact. Ultimately, these findings may help inform the development of accessible data-driven tools for personalized T2D risk prediction and early intervention.

## 2 Subjects

### 2.1 Discovery dataset

The discovery dataset used for this study comprised participants from the FGENTCARD Consortium, a non-European (Lebanese) population, a subgroup of the larger CARDIoGRAMplusC4D consortium [23], [24], [25]. All individuals volunteered and gave written consent. The Institutional Review Board (IRB) at the Lebanese American University approved the study, ensuring adherence to the Helsinki Declaration. 3,546 genotyped subjects were included (Supplementary Figure 1), split into T2D patients and matched control subjects. T2D cases were defined based on clinical documentation in the patients’ medical records.

Controls were defined as individuals without a diagnosis for T2D, confirmed through medical records. Participants were included if they provided comprehensive health data, blood samples for biomarker and genomic analyses, and completed the required questionnaires. Exclusion criteria included participants with missing genotype data. All participants completed lifestyle questionnaires that captured information on health behaviors and medical conditions including T2D, hypertension, CVD, and hyperlipidemia. Medication details were also collected from their medical records. Blood samples were collected, and two blood collection tubes were used to separate plasma and serum for DNA extraction, lipid profile analysis, and other biomarker tests. Data was collected between 2007 and 2016 [25]. Age of onset for T2D was obtained from the medical record of each patient after securing IRB approval of the study in addition to a signed consent form from each patient. Participants were eligible for inclusion if they were 18 years or older, provided written informed consent, and were admitted to one of the recruitment centers. There was no patient or public involvement in this study's design, conduct, reporting, interpretation, or dissemination.

### 2.2 Clinical and genomics data in the discovery dataset

Clinical features were collected through self-reported questionnaires completed by participants at the time of recruitment and patients’ medical records. The data included demographic variables (age, gender), anthropometric measures (weight, BMI, height), and various biomarkers (glucose, triglyceride, LDL, HDL, Total Cholesterol). It also includes information on comorbidities (hyperlipidemia, hypertension, cardiovascular disease (CVD), family history (Fx) of diseases, and lifestyle factors (smoking).

Genomic DNA was extracted using the phenol extraction method. Genomic data consisted of 2,414 SNPs previously associated with T2D, curated from published genome-wide association studies (GWAS) and literature [25], [26], [27], [28], [29], [30], [31]. Of these, 449 SNPs were genotyped in the discovery dataset using two platforms: the Illumina Human610-Quad BeadChip and the Illumina Human660W-Quad BeadChip. Quality control measures were applied using PLINK 1.9 software to ensure robust analysis. SNPs were excluded based on the following criteria: [1] minor allele frequency (MAF) < 1 %, [2] Hardy–Weinberg Equilibrium p-value < 0.001, and [3] missing genotype call rate > 20 %. Although the 20 % threshold is more lenient than the typical 5–10 % used in genome-wide studies, it was chosen to retain SNPs of known biological relevance within a targeted panel while still ensuring sufficient call rate for analysis. Individual-level QC included removal of participants with call rates < 95 %. After quality control, 135 SNPs remained. These were further filtered to select one SNP per gene, prioritizing those with the strongest evidence of association from GWAS, resulting in a final set of 83 SNPs. This strategy was employed to reduce redundancy, minimize multicollinearity, and improve model interpretability, while maintaining relevance to known T2D-associated loci.

### 2.3 Replication dataset: UK Biobank

The UK Biobank dataset (N = 31,620; application number 64823), predominantly includes individuals of European descent, was used for external validation and replication of the ML models. This validation step supports the generalizability of the models and their performance in an independent population distinct from the discovery dataset. T2D cases were identified using the International Classification of Diseases, Tenth Revision (ICD-10) code E11. Control subjects were selected based on the absence of any T2D diagnosis and were stratified to match the gender distribution of the T2D cases, thereby minimizing sex-related confounding. To ensure population homogeneity and reduce population stratification bias, all subjects included in the analysis were of confirmed British ancestry.

## 3 Methods

### 3.1 Statistical analysis

Statistical analysis was conducted using the R package (version 4.2.2). Continuous variables were evaluated using One-Way ANOVA, while categorical variables were compared using the Chi-squared test.

### 3.2 Study design

The sample size for this study was determined based on the availability of genotyped participants in the FGENTCARD cohort (N = 3,546) and the UK Biobank replication dataset (N = 31,620) (Supplementary Figure 1). The study size was considered adequate to train and evaluate machine learning models. The large sample size in the UK Biobank further ensured the generalizability of the findings.

ML algorithms were evaluated in both datasets, including RF, DT, SVM, LDA, LR, and GBM using R software. The SVM model was trained using a linear kernel (svmLinear) implemented via the caret package in R. These models provide a diverse representation of learning paradigms (e.g., linear vs. non-linear, ensemble vs. individual learners), suited for comprehensive performance evaluation across clinical and genetic predictors. The discovery dataset, FGENTCARD (N = 3,546), was used for model development and internal validation, while the replication dataset, UK Biobank (N = 31,620), was used to evaluate the models’ generalizability independently. The study adheres to the Transparent Reporting of a multivariable prediction model for Individual Prognosis Or Diagnosis (TRIPOD) guidelines with a completed checklist provided in Supplementary Table 1 to ensure methodological transparency and reproducibility.

### 3.3 Modeling approaches

The performance of the models was assessed using three complementary approaches: i) clinical and demographic features only: models were trained on 17 clinical and demographic features; ii) a combination of clinical, demographic and genomics features: models were evaluated using a combination of the same 17 clinical features plus 83 SNPs (47 of the 83 SNPs were genotyped in the UK Biobank); iii) Age-specific analyses: models were trained separately for two age groups, individuals > 55 years and individuals ≤ 55 years, using both feature sets: the clinical data alone and the combination of clinical and genomic data. The age cutoff of 55 years is not an arbitrary cutoff. It was selected based on epidemiological and biological relevance. According to the International Diabetes Federation (IDF) Atlas (2021), the 55–59 age group represents the peak prevalence of T2D globally, projected to increase significantly by 2045 [32]. This stratification also aligns with previous UK Biobank studies, which categorize individuals based on this age threshold (≤55 years vs. >55 years) [33]. Sensitivity analysis using alternative cutoffs (60 and 65 years) was also conducted to evaluate model performance stability (Supplementary Figure 2). The 55-year threshold yielded superior predictive performance, particularly in the younger age group (≤55 years). Therefore, this cutoff was used as it maximized model performance while maintaining biological and clinical relevance.

### 3.4 Data pre-processing

Data preprocessing aimed to ensure data integrity and model compatibility. The goal was to produce an accurate and valuable final dataset for subsequent data mining algorithms. Records with missing key features were excluded, as not all participants had complete clinical or laboratory data [34]. Records with missing values in key variables (e.g., age, BMI, T2D status, or prioritized SNPs) were excluded. Approximately 12 % of subjects were removed from the discovery dataset and 13 % from the UK Biobank dataset. Only complete cases were retained to ensure consistency and reliability in model training and evaluation.

Class imbalance, where one outcome occurs more frequently than another, is a common challenge in ML applications. In the discovery dataset, the prevalence of T2D cases was 38.3 % compared to 61.7 % for controls, potentially biasing the models towards the majority class [35], [36]. To address this, class weighting was applied during model training. Weights were calculated as the inverse of class proportions and incorporated into the model training process using the caret package. This approach adjusted model optimization to focus more on the minority class, thereby improving sensitivity without sacrificing overall sample size. It was applied consistently across all models to ensure comparability and fairness in learning. This strategy allowed for equitable learning without excluding any observations. [35], [36].

### 3.5 Model development and hyperparameter tuning

All analyses were conducted using R (version 4.2.2), with model training and evaluation performed using the caret package, (an open-source software for machine learning)*.* The preprocessed discovery dataset was randomly divided into training (75 %) and testing (25 %) sets. The test set remained untouched during training to ensure unbiased evaluation. Model optimization was performed using 10-fold cross-validation, repeated 3 times, to improve model stability and minimize overfitting. Each model was trained on nine subsets and validated on the remaining one, cycling through all combinations. Hyperparameter tuning was performed using grid search to identify optimal parameters based on performance metrics, primarily accuracy. The final models were then evaluated on the independent test set.

To assess generalizability, the UK Biobank dataset was used for external validation. All three modeling configurations- clinical-only, clinical plus genomics, and age-stratified models- were replicated in this dataset.

### 3.6 Model evaluation

Model performance was evaluated based on the multiple metrics derived from the confusion matrix: accuracy, precision, F1-score, recall, Matthews Correlation Coefficient (MCC), and Cohen’s Kappa. The F1-score provides a balanced mean of precision and recall, balancing sensitivity and specificity while MCC captures overall binary classification quality, accounting for true and false positives and negatives, yielding values ranging from ‐ 1 (total disagreement) to + 1 (positive prediction). Cohen’s kappa quantifies inter-rater reliability adjusting for chance-level agreement. Receiver Operating Characteristic (ROC) curves and corresponding area under the curve (AUC) were computed to assess model discrimination capability. Given the class imbalance in the dataset, Precision-Recall (PR) curves and PR-AUC were also calculated, as these metrics are more informative than ROC-AUC in skewed datasets. Model calibration (the agreement between predicted probabilities and actual outcomes), was assessed using calibration plots and the Brier score (the mean squared difference between predicted probabilities and the observed outcome). All ML models were implemented in R (RandomForest, rpart, tree, and gbm) with performance metrics computed using MLmetrics and PRROC.

### 3.7 Variable importance

Variable importance was assessed across all six ML models (RF, SVM, LR, GBM, LDA, and DT) to identify the most influential predictors of T2D risk. For RF, LR, DT, and GBM models, the VIP() function was utilized to quantify feature importance. For SVM, feature importance was derived from weight vectors ordered by descending weight. For LDA, scaling and coefficient values were analyzed to infer predictor contribution. To better understand age-specific predictors, variable importance was stratified by age group (≤55 years vs. >55 years) and compared across models trained with clinical features alone and those combining clinical and genomic data. To evaluate the overall importance of each feature, a composite weighted variable importance score was calculated (by aggregating feature ranks across the six ML models) to summarize the overall impact of each feature across models. Within each model, features were assigned a reverse-rank score from 10 (most important) to 1 (least significant), with higher scores indicating higher model-specific importance. These scores were then aggregated across the six models generating a total importance weight per feature. This scoring approach enables cross-model comparison of predictors contributing most strongly to T2D risk stratification (i.e., clinical alone vs. clinical + genomic).

### 3.8 Polygenic risk score calculation

A trans-ancestry polygenic risk score (PRS) for T2D was calculated for both the discovery dataset and UK Biobank validation dataset. The PRS was for each individual *i* was calculated as:

$PRS_{i}=\sum_{j=1}^{m}G_{ij}\times \;\log(OR_{j})$

where G*ij* represents the genotype dosage (0, 1, or 2) for individual *i* at SNP *j*, and *m* is the total number of SNPs common to both datasets contributing to the PRS. The PRS was standardized (mean = 0, SD = 1) before inclusion in model training. The PRS was integrated alongside clinical predictors to evaluate its incremental contribution to T2D risk prediction. The PRS was calculated using R (version 4.2.2).

### 3.9 Model deployment and accessibility

The ML pipeline and trained prediction model are made publicly available to promote reproducibility and to support clinical and research applications. All R scripts for data preprocessing, model training, and evaluation are hosted on GitHub at https://github.com/cynthia-hg/T2D-ML-Prediction. To facilitate clinical and research use, the final model was deployed as an interactive Shiny web application on Hugging Face Spaces (https://huggingface.co/spaces/T2D-Research-Team/T2D_ML). This interface allows users to input clinical and genomic features and obtain risk predictions. The deployed app uses the RF model trained on clinical and genomic data, chosen for its high accuracy (0.687), strong overall performance, and compatibility with the Shiny environment. The GitHub repository includes the full codebase and trained model objects for all evaluated algorithms (https://github.com/cynthia-hg/T2D-ML-Prediction/tree/main/trained_model), enabling replication or alternative deployments.

## 4 Results

### 4.1 General characteristics of the study population

Table 1 displays the general characteristics of the study population, comprising 2,187 controls and 1,359 patients with T2D. Individuals with T2D were generally older and exhibited higher levels of triglycerides and glucose, along with lower levels of HDL, LDL, and total cholesterol compared to those without T2D. Moreover, they were more likely to have hyperlipidemia, hypertension, CVD, and Fx. T2D compared to those without T2D (controls).

**Table 1:** Descriptive table of the study population.

**Variables**	**Controls (2,187)**	**Cases (1,359)**	**Total (3,546)**	**p. value**
**Age**	62.45 (0.25)	63.43 (0.27)	62.83 (0.19)	< 0.01
**Weight**	77.30 (0.31)	77.07 (0.38)	77.21 (0.24)	0.65
**BMI**	28.06 (0.10)	28.22 (0.13)	28.13 (0.08)	0.32
**Height**	165.95 (0.21)	165.27 (0.26)	165.69 (0.17)	0.05
**Glucose**	101.19 (0.54)	157.46 (2.01)	119.70 (0.91)	< 0.01
**Triglyceride**	165.37 (2.12)	191.03 (3.49)	175.17 (1.88)	< 0.01
**LDL cholesterol**	119.24 (0.94)	105.82 (1.10)	114.13 (0.72)	< 0.01
**HDL cholesterol**	43.90 (0.30)	39.83 (0.33)	42.34 (0.22)	< 0.01
**Total cholesterol**	195.25 (1.11)	180.60 (1.32)	189.64 (0.86)	< 0.01
**Gender**				0.84
**Female**	757 (34.6 %)	475 (35.0 %)	1232 (34.7 %)	
**Male**	1430 (65.4 %)	884 (65.0 %)	2314 (65.3 %)	
**Hyperlipidemia**				< 0.01
**No**	1291 (59.3 %)	587 (43.3 %)	1878 (53.2 %)	
**Yes**	886 (40.7 %)	769 (56.7 %)	1655 (46.8 %)	
**Hypertension**				< 0.01
**No**	1154 (52.8 %)	427 (31.4 %)	1581 (44.6 %)	
**Yes**	1032 (47.2 %)	931 (68.6 %)	1963 (55.4 %)	
**CVD**				< 0.01
**No**	945 (43.3 %)	381 (28.1 %)	1326 (37.5 %)	
**Yes**	1237 (56.7 %)	976 (71.9 %)	2213 (62.5 %)	
**Fx. Hyperlipidemia**				0.85
**No**	1368 (63.3 %)	849 (63.0 %)	2217 (63.2 %)	
**Yes**	793 (36.7 %)	499 (37.0 %)	1292 (36.8 %)	
**Fx. Hypertension**				0.22
**No**	900 (41.8 %)	591 (43.9 %)	1491 (42.6 %)	
**Yes**	1254 (58.2 %)	755 (56.1 %)	2009 (57.4 %)	
**Fx. T2D**				< 0.01
**No**	1190 (55.1 %)	390 (28.9 %)	1580 (45.0 %)	
**Yes**	968 (44.9 %)	960 (71.1 %)	1928 (55.0 %)	
**Fx. CVD**				0.28
**No**	840 (38.9 %)	550 (40.7 %)	1390 (39.6 %)	
**Yes**	1319 (61.1 %)	800 (59.3 %)	2119 (60.4 %)	
**Smoking**				0.52
**No**	763 (35.7 %)	485 (36.8 %)	1248 (36.1 %)	
**Yes**	1373 (64.3 %)	833 (63.2 %)	2206 (63.9 %)	

Data are mean values ± SE (standard error). Glucose (mg/dL). BMI: Body mass index (Kg/m^2^). Total cholesterol (mg/dL). LDL cholesterol (mg/dL). HDL cholesterol (mg/dL). Triglyceride (mg/dL). Fx.: Family History. Cases: patients with T2D. T2D: type 2 diabetes. P-values obtained using *t*-test for continuous variables and chi-squared for categorical variables.

### 4.2 Model performance based on only clinical data

In the discovery dataset, models trained using only 17 clinical features demonstrated varied performance across metrics (Table 2). The GBM model achieved the highest AUC (72.460 %), followed closely by LDA (72.170 %) and LR (72.089 %) (Supplementary Figure 3). GBM also showed the highest recall (0.949) and F1-score (0.775), highlighting its strong capability in correctly identifying T2D cases. While GBM excelled in sensitivity-related metrics, LDA and RF achieved the highest accuracy (0.687), and LR demonstrated the highest precision (0.772). Across all models, clinical variables such as Fx.T2D, hypertension, and CVD were consistently ranked among the top predictors (Table 3**,** Supplementary Figure 4).

**Table 2:** Performance comparison of the six prediction models based on clinical data and clinical + genomic data.

	Model	Accuracy	Precision	Recall	F1_score	MCC	Kappa	AUC %	Brier score	PR_AUC
**Clinical data**										
1	RF	0.687	0.701	0.859	0.772	0.304	0.288	71.811	0.204	0.601
2	LR	0.666	0.772	0.650	0.706	0.332	0.325	72.089	0.215	0.603
3	GBM	0.659	0.654	0.949	0.775	0.224	0.164	72.460	0.203	0.594
4	SVM	0.676	0.714	0.793	0.752	0.294	0.291	71.957	0.204	0.604
5	DT	0.670	0.690	0.844	0.759	0.263	0.250	65.206	0.218	0.520
6	LDA	0.687	0.717	0.814	0.762	0.314	0.308	72.170	0.203	0.604
**Clinical +SNPs data**										
1	RF	0.687	0.718	0.810	0.762	0.315	0.310	72.317	0.203	0.599
2	LR	0.668	0.773	0.655	0.709	0.336	0.330	71.980	0.218	0.599
3	GBM	0.667	0.661	0.947	0.778	0.248	0.188	73.325	0.200	0.609
4	SVM	0.671	0.719	0.765	0.742	0.291	0.290	71.924	0.204	0.602
5	DT	0.670	0.690	0.844	0.759	0.263	0.250	65.206	0.218	0.520
6	LDA	0.680	0.728	0.770	0.748	0.312	0.311	72.118	0.206	0.602

LR = logistic regression, RF = random forest, SVM = support vector machine, LDA: linear discriminant analysis, DT: decision tree, GBM: Gradient Boosting Machines, MCC = Matthews Correlation Coefficient, and Kappa = Cohen’s kappa score. AUC: Area under the curve. PR: Precision-Recall.

**Table 3:** The top model-specific VIPs based on clinical data and clinical + genomics data.

**Rank**	**RF**	**SVM**	**LDA**	**LR**	**GBM**	**DT**
**Clinical data**						
1	Triglyceride	Fx.T2DYes	Fx.T2DYes	Fx.T2DYes	Fx.T2DYes	Fx.T2D
2	LDL	HypertensionYes	HypertensionYes	HypertensionYes	HypertensionYes	Hypertension
3	Totalcholesterol	CVDYes	CVDYes	CVDYes	HDL	HDL
4	HDL	HyperlipidemiaYes	HyperlipidemiaYes	HyperlipidemiaYes	LDL	Triglyceride
5	Height	Fx.CVDYes	Fx.HypertensionYes	Fx.HypertensionYes	CVDYes	Total.Cholesterol
6	BMI	BMI	Fx.CVDYes	Fx.CVDYes	Triglyceride	LDL
7	Age	Fx.HypertensionYes	GenderMale	HDL	Age	Fx.Hypertension
8	Weight	Weight	Fx.HyperlipidemiaYes	LDL	HyperlipidemiaYes	Age
9	Fx.T2D	Height	Smoking.Yes	Triglyceride	Weight	CVD
10	Hypertension	HDL	BMI	GenderMale	Fx.CVDYes	Gender
**Clinical + SNPs data**						
1	Triglyceride	Fx.T2DYes	rs12137794TT	Fx.T2DYes	Fx. T2D	Fx. T2D
2	LDL	HypertensionYes	rs7202877TT	HypertensionYes	Hypertension	Hypertension
3	HDL	rs9275595TT	Fx.T2DYes	CVDYes	Total cholesterol	Total cholesterol
4	Fx.T2D	rs2075650GG	rs3132946GG	HyperlipidemiaYes	Age	LDL cholesterol
5	Height	rs7756992GG	rs7202877GT	Fx.HypertensionYes	HDL cholesterol	Age
6	Age	HyperlipidemiaYes	rs3132946AG	rs7903146TT	LDL cholesterol	HDL cholesterol
7	Totalcholesterol	CVDYes	rs3130501AG	rs7756992GG	CVD	Fx. CVD
8	BMI	rs7202877TT	rs3130501GG	Fx.CVDYes	Height	Fx. Hypertension
9	Weight	rs9275595CT	HypertensionYes	rs7903146TC	Hyperlipidemia	CVD
10	Hypertension	rs7202877GT	rs9275595TT	HDL	Triglyceride	Triglyceride
11	rs7903146	rs7903146TT	rs7756992GG	rs8042680AC	rs940904	Gender
12	rs7756992	rs3130501GG	CVDYes	rs10965243GA	Weight	rs1215470
13	CVD	rs3130501AG	rs2075650GG	rs9275595TT	BMI	Weight
14	rs1260326	rs2395163TT	rs2943641TC	rs1260326TT	rs7903146	BMI
15	rs10906115	rs2246012TT	HyperlipidemiaYes	rs7756992GA	rs940904	Gender

VIPs: variable importance in projection, LR = logistic regression, RF = random forest, SVM = support vector machine, LDA: linear discriminant analysis, DT: decision tree, GBM: Gradient Boosting Machines.

### 4.3 Model performance based on clinical and genomics data

Incorporating genomic data (17 clinical features + 47 SNPs), yielded modest but consistent gains in model performance. For example, the AUC of the GBM model increased from 72.460 % to 73.325 %, indicating a modest improvement in discriminative ability, its recall reached 0.949 and the F1-score 0.778. LR achieved the highest precision (0.773), while RF attained the highest accuracy (0.687). Together, these results demonstrate that integrating genomic data adds predictive value beyond clinical variables alone.

Overall, GBM was the best-performing model across both clinical-only and clinical + genomic scenarios, consistently achieving the highest AUC and F1-score.

### 4.4 Variable importance analysis

Using clinical features alone (Table 3), Fx.T2D and hypertension remained the most influential predictors across all six models. When genomic data were included, Fx.T2D, hypertension, and CVD remained the top-ranked variables across most models (Supplementary Figure 4**)**. Key SNPs such as rs7903146 and rs7756992 were frequently among the top-ranked features, particularly in SVM, LDA, and LR. However, tree-based models (RF, GBM, and DT) remained dominated by clinical predictors even after genomic integration (Table 3).

### 4.5 Age-stratified performance in the discovery dataset

Model performance was assessed separately for individuals older than 55 years and those 55 years or younger (Supplementary Table 2). Across accuracy, precision, recall, and F1-score, models performance was consistently superior in the younger age group (≤55). For instance, GBM achieved an accuracy of 0.719 in the ≤ 55 group compared to 0.625 in the > 55 group. Similarly, RF achieved an accuracy of 0.730 in the ≤ 55 group versus 0.672 in the > 55 group and AUC values also improved in the ≤ 55 group, particularly for DT and LDA.

Adding genomic data, the performance advantage in younger individuals became even more evident. Across most metrics, models performed better in the ≤ 55 age group. For instance, GBM’s accuracy increased to 0.730 in the younger group compared to 0.649 in the older group, and RF improved from 0.730 vs. 0.672. Among younger individuals (≤55), GBM achieved the highest recall (0.937) and F1-score (0.831), while RF had the highest AUC (73.016 %) and accuracy (0.730), underscoring the strength of both models in early risk prediction in younger adults.

Age-stratified variable importance patterns diverged with age. While Fx.T2D remained the top-ranked predictor across all models, in older individuals traditional clinical measures such as LDL, triglycerides, HDL, and hypertension dominated. In contrast, younger individuals exhibited stronger contributions from genetic variants, notably SNPs like rs9275595 and rs7756992, which consistently ranked highly across the models (Supplementary Table 3**,** Supplementary Figure 4). These findings suggest that genomic markers play a more prominent role in T2D prediction among younger adults, potentially enabling earlier identification of at-risk individuals before traditional risk factors fully emerge.

### 4.6 Model performance in the UK Biobank

In the UK Biobank, overall model performance was higher than in the discovery dataset (Supplementary Table 4). Using clinical features alone, GBM, RF, and LR achieved the highest AUCs (91.503, 91.426, and 91.003 %, respectively). When 47 SNPs were added to the clinical features, GBM achieved the highest AUC (91.769 %), followed by LR (91.233) and RF (91.172). Recall and F1-score metrics showed similar improvements across models.

In the UK Biobank dataset, GBM again achieved the highest AUC and recall, while RF showed the highest accuracy. This consistency across datasets supports the robustness of the top-performing models.

### 4.7 Variable importance analysis in UK Biobank models

The UK Biobank models include clinical variables such as hypertension, total cholesterol, and Fx. T2D consistently ranked among the most important features across all machine learning models (Supplementary Table 5**,** Supplementary Figure 4). In models incorporating both clinical and genomics data, well-known SNPs such as rs7903146 (*TCF7L2*) and rs2943641 (*IRS1*) emerged as significant contributors, particularly in GBM, SVM, LR, and LDA models. Despite integrating genomics data, clinical factors like hypertension, cholesterol, and Fx.T2D remained dominant predictors, underscoring their critical role in T2D risk assessment. Including genomics data significantly improved the predictive performance of the models, with SNPs such as rs2943641 and rs7903146 consistently ranking among the top 10 variables in most models, reinforcing their relevance in T2D prediction.

### 4.8 Impact of age on model performance and key predictors in the UK Biobank

In the UK Biobank dataset, all six algorithms performed better among individuals aged ≤ 55 compared to those > 55 (Supplementary Table 6). With clinical data alone, GBM achieved the highest AUC in the younger group (92.028), compared to 91.282 in the older group. Both AUC and accuracy were consistently higher across all models in the ≤ 55 group.

The inclusion of genomic data further amplified this performance gap. In the ≤ 55 group, GBM’s AUC rose from 92.03 % (clinical only) to 92.43 % (clinical + genomic), whereas in the > 55 group it increased from 91.28 % to 91.46 %. These findings suggest that SNP integration confers the greatest incremental benefit for early risk prediction in younger adults.

Across both age groups, clinical features such as hypertension, total cholesterol, Fx.T2D, and BMI consistently ranked among the top predictors when only clinical data were used (Supplementary Table 4). However, with the addition of genomic data, key SNPs such as rs7903146 and rs2943641 emerged as strong predictors, especially in the younger group.

### 4.9 Feature importance shifts with age and genomic integration

Analyzing feature importance across models stratified by age and data type in the Discovery dataset revealed notable shifts in variable ranking when SNPs were incorporated (Supplementary Figure 3). In the younger age group (≤55 years), *rs9275595*TT emerged as a top-ranked genomic feature, appearing in 3rd position when genomic data were included, surpassing traditional clinical variables such as BMI and triglycerides, which were ranked 5th and 7th, respectively, in the clinical-only model. Similarly, rs7756992 and rs3130501 entered the top 10 upon SNP addition, replacing features like height and LDL. Conversely, for individuals > 55 years, clinical predictors like LDL and triglycerides remained consistently dominant, although rs13342692 and rs7903146 rose to mid-tier positions (ranks 5–6), reflecting a modest but noticeable genomic influence in older adults. Notably, across all age groups, Fx.T2D and hypertension retained their dominant status as the top two predictors, confirming their central role in T2D risk prediction. Genomic integration not only elevated genomic markers into the top ranks, particularly in younger individuals, but also slightly demoted mid-ranked clinical features, illustrating how genomic data shifts the relative influence of features in the model.

In the UK Biobank analysis, incorporating genomic data led to noticeable shifts in the feature importance rankings across age groups. For individuals > 55 years, Fx.T2D moved up from 4th position in the clinical-only model to 2nd in the clinical + genomic model, overtaking features such as triglycerides and BMI. Additionally, SNPs like rs2943641, rs7903146, and rs7756992 entered the top 10, displacing traditional predictors such as smoking and gender. For individuals aged ≤ 55, although hypertension remained the top feature across both data types, Fx.T2D rose from rank 6 to rank 5 with SNP integration, while rs2943641 entered at rank 9. These shifts suggest genomic data provides added discriminative power without displacing core clinical features. When considering the full population, rs7903146 and rs2943641 ranked within the top 10 predictors only after SNPs were included, reaffirming their established roles in T2D susceptibility. Across all age groups, hypertension, total cholesterol, and hyperlipidemia retained dominant positions regardless of SNP inclusion, underscoring their foundational relevance. Notably, the relative importance of SNPs was more evident in the younger group, where they replaced several clinical variables in the top 10, highlighting the potential of genetic screening to augment early risk detection when conventional risk markers may not yet be clinically elevated.

### 4.10 Model calibration and discrimination performance

Model calibration and probability reliability were assessed using Brier scores and calibration curves (Table 1, Supplementary Tables 2 and 4, and Supplementary Figure 6). To account for class imbalance, PR-AUC was also calculated to measure classification performance. Overall, all six models demonstrated good calibration (low Briar scores) and strong discrimination (high ROC-AUC and PR-AUC) performance, confirming their robustness in T2D risk prediction.

### 4.11 Polygenic risk score analysis

We assessed the integration of a trans-ancestry polygenic risk score (PRS) alongside clinical features in both the discovery dataset and UK Biobank. In the discovery dataset, Clinical + PRS models showed variable performance across algorithms and age groups. Notably, SVM achieved the highest AUC (74.3 %) for individuals ≤ 55 years, suggesting potential value of PRS for early risk prediction (Supplementary Table 7). However, overall, PRS-enhanced models performed comparable to clinical + targeted-SNP models.

In the UK Biobank, PRS integration supported strong model performance across age groups. GBM achieved the highest AUC while SVM yielding the highest accuracy in clinical + PRS integration. These findings indicate that while PRS contributed meaningful genetic information, its added predictive value beyond clinical and targeted SNP data was modest in large, well-phenotyped cohorts.

## 5 Discussion

Traditional statistical approaches like logistic regression have been widely used to develop disease risk scores and prediction models. However, these linear models may oversimplify complex nonlinear relationships among features, potentially overlooking subtle but clinically important patterns [37]. In contrast, machine learning algorithms, particularly those capable of modeling non-linear relationships, have consistently outperformed linear models in chronic disease risk prediction, including T2D [3], [37], [38], [39], [40], [41].

This study aimed to evaluate whether adding genomic data to key clinical and epidemiological variables could enhance the performance of ML -based T2D risk models. Six ML algorithms were applied to a discovery dataset of 3,546 subjects using clinical data alone (17 features) or those features plus 47 SNPs. The addition of genomic data modestly improved model performance metrics across models, affirming the complementary value of genomic data for refining T2D risk prediction.

The composition of our study population was designed to include subjects with and without T2D. The discovery dataset exhibited a class imbalance of 61.7 % non-T2D and 38.3 % T2D cases. Class imbalance can bias ML models towards the majority class and affect metrics (e.g., precision-recall and false-positive rates), particularly when applied to routine screening. This results in poor classification performance for the minority class [35], [36]. To preserve the real-world class distribution (the full dataset), class weights were applied during training, thereby allowing the algorithms to emphasize the underrepresented T2D class. External validation using the UK Biobank (N = 31,620) provided an independent evaluation of model generalizability across ancestrally distinct populations. Despite demographic and genetic differences, this independent evaluation was intended to assess the robustness and transferability of clinical and genomic predictors, which is an essential step toward developing broadly applicable precision medicine tools.

In the UK Biobank replication (N = 31,620), all six models achieved high performance with combined clinical and genomic data (AUC > 91 %). GBM, LR, and RF achieved the highest AUCs (91.769 %, 91.233 %, and 91.172 %, respectively). Across every model, Fx. T2D, hypertension, and cholesterol emerged as the most dominant features highlighting their role in risk stratification. Upon integrating genomic features, T2D-susceptibility loci like rs7903146 (*TCF7L2*), rs2943641 (*IRS1*), and rs7756992 (*CDKAL1*) consistently ranked among top predictors especially in linear models (SVM, LR, and LDA models). Tree-based models like RF and GBM remained primarily driven by clinical variables.

All implicated SNPs map in or near genes with well-characterized roles in T2D pathogenesis reinforcing their mechanistic relevance to both insulin resistance and β-cell dysfunction. *TCF7L2* (rs7903146, intronic) regulates insulin secretion and β-cell function via the canonical Wnt/β-catenin pathway. *IRS1* (rs2943641, ∼5 kb upstream) is central to insulin receptor signaling and peripheral insulin sensitivity, while *CDKAL1* (rs7756992, intronic) regulates proinsulin processing and β-cell energy metabolism. In age-stratified analyses, variants near *HLA class II* region (rs9275595 within locus*)* and *TCF19* (rs3130501 ∼10 kb upstream) were prominent in individuals ≤ 55 years, suggesting their respective roles in immune modulation and β-cell survival mechanisms in early-onset disease. *SLC16A11* (rs13342692, intronic) influences hepatic lipid handling supporting a role in lipid metabolism and hepatic insulin resistance in later-onset T2D [42], [43], [44], [45], [46], [47]. These dynamics highlight how integrating genomic markers can refine understanding of the shifting molecular drivers that underlie T2D as a continuous progressive disorder.

Age-stratified analyses consistently demonstrated superior performance in individuals aged ≤ 55 compared to > 55, in both the discovery and UK Biobank datasets. In the UK Biobank, using clinical data alone, GBM’s AUC increased from 91.282 % in the > 55 group to 92.028 % in the ≤ 55 group. Integrating genomic data further improved the AUC in the younger group to 92.432 %. These findings suggest greater benefit from genomic integration in early-onset T2D prediction and emphasize the enhanced predictive power of genomic makers in younger individuals, likely due to the absence of overt metabolic risk markers at early disease stages. In contrast, the modest accuracy observed in the older age group (> 55 years) may reflect increased heterogeneity in health status resulting from cumulative lifestyle exposures, environmental factors, and subclinical disease. Over decades, older individuals may vary widely in physiological resilience, with some exhibiting metabolic profiles similar to younger adults, while others show more advanced risk phenotypes. This variability likely contributes to reduced model discriminability. To enhance prediction in older populations, future models could incorporate longitudinal data, richer lifestyle indicators (e.g., physical activity, diet, and stress), or additional omics layers (e.g., metabolomics, proteomics) to better capture the complexity of T2D risk trajectories in aging populations.

The use of 55 years as an age cutoff in this study reflects a clinically meaningful and widely accepted stratification point in T2D research. Epidemiological data indicate a marked rise in T2D incidence and prevalence beyond this age, with large cohort studies showing that the 10-year risk of progression increases notably after 55 [48]. This threshold aligns with established conventions recognizing 55 as the transition between early and late middle age [32] and is consistent with stratification approaches used in prior UK Biobank studies [33]. Sensitivity analyses using alternative cutoffs (60 and 65 years) confirmed that the 55-year threshold provided optimal model performance and stability, particularly for identifying risk in younger individuals (i.e., ≤ 55 years) (Supplementary Figure 2).

To further evaluate model reliability, calibration was assessed using Brier scores, calibration curves, and PR-AUC. Across both discovery and replication cohorts all six algorithms delivered well-calibrated probabilities and strong discrimination. PR-AUC values were notably higher with the inclusion of genomic features especially in younger individuals ≤ 55 years, indicating enhanced sensitivity for identifying true positives, which is vital for early intervention strategies.

Consistently across the discovery and replication datasets in younger individuals (>55 years), Fx. T2D, hypertension, and several key T2D-associated SNPs (e.g., rs7903146, rs7756992, and rs2943641) dominated the variable importance rankings, displacing mid-ranking clinical features such as BMI, triglycerides, and LDL, suggesting their relative importance in early-onset disease. While previous studies have reported that including common genetic variants associated with T2D only marginally improved the prediction of future T2D compared to clinical risk factors alone [49], ML models offer an advantage by detecting complex, nonlinear interactions, even subtle signals from diverse data sources. In younger cohorts, where clinical markers often remain within normal ranges, genetic predisposition emerges as a powerful predictor. Incorporating SNPs therefore unearths latent T2D risk in asymptomatic individuals, supporting proactive genetics-informed screening and personalized prevention.

Validated in the UK Biobank, these findings suggest that genomic markers exert a greater influence on risk prediction in individuals ≤ 55 years. Age-stratified modeling thus not only improves overall predictive performance but may also uncover distinct pathophysiological drivers: in younger individuals, SNPS uncover latent genetic risk before clinical abnormalities emerge; whereas in older individuals, their contribution is more modest amid established metabolic dysfunction. Clinically, these results support early genetics-informed screening and tailored lifestyle changes, frequent monitoring, and targeted risk reduction strategies at the stages when they can be most effective.

The analysis of variable importance across both datasets highlights how integrating SNP data subtly reshapes the relative contributions of features in T2D prediction. In the discovery and UK Biobank cohorts, traditional clinical predictors such as Fx.T2D, hypertension, and cholesterol-related variables remained dominant, underscoring their continued value in risk stratification. In the discovery dataset, these SNPs displaced mid-ranked clinical variables like BMI, triglycerides, and LDL, suggesting that genomic features may carry more predictive weight in individuals who have not yet developed overt metabolic disturbances. A similar pattern was observed in the UK Biobank, where SNPs gained prominence in the younger subgroup while complementing, rather than replacing, key clinical features. In older individuals (> 55 years), clinical predictors retained more stable rankings, although SNPs still entered the mid-tier of importance, reflecting a more modest but detectable contribution. These shifts confirm that genomic information can enhance model performance by identifying risk contributors, especially relevant in younger adults, where traditional markers alone may not capture the whole picture. Importantly, the consistent emergence of Fx.T2D, hypertension, and top-ranked SNPs across models and datasets reinforces the robustness and generalizability of these predictors in T2D risk modeling particularly in early-onset cases while preserving the predictive value of core clinical factors.

A trans-ancestry PRS was integrated with clinical features in both the discovery and UK Biobank cohorts to evaluate its incremental value. In the discovery dataset, clinical + PRS models showed variable performance across algorithms. SVM achieved the highest AUC (74.3 %) in the ≤ 55 group. In GBM and RF models, the PRS ranked among the top predictors in the younger subgroup (≤ 55 years). This highlights the potential of PRS in early risk prediction where traditional clinical indicators may not fully capture risk. In the UK Biobank, PRS integration contributed to robust model performance across age groups, with GBM generally achieving the highest AUC and SVM demonstrating the highest accuracy in clinical + PRS models. Although the PRS ranked highly in variable importance analyses, particularly in younger individuals, its incremental predictive value beyond clinical features and selected SNPs was modest overall in this study. This aligns with previous research that showed that while PRS may offer limited additional AUC gain when strong clinical predictors are present, it can meaningfully enhance risk prediction, reclassification, and early detection especially in younger individuals or lower risk subgroups [50], [51]. Future research could explore optimizing trans-ancestry PRS models and integrating them into multi-layered risk tools to further support early detection and precision prevention strategies.

Previous studies have demonstrated that the genetic architecture of T2D varies by age at diagnosis, with specific loci demonstrating stronger associations with early-onset forms of the disease [52]. This age-dependent genetic heterogeneity underscores the value of age-stratified modeling to uncover distinct pathophysiological mechanisms underlying T2D development and supports precision medicine approaches. By integrating genetic and clinical predictors within age-specific risk models, it is possible to refine screening strategies and optimize early intervention efforts in high-risk younger individuals, especially those with strong family histories of T2D or borderline clinical indicators. Embedding these models into routine clinical workflows could help prioritize intensive screening, especially for individuals with significant clinical risk factors (e.g., family history, hypertension), and a genetic predisposition marked by SNPs such as rs7903146 and rs7756992. Future studies could explore incorporating these models into public health initiatives or T2D prevention frameworks. In doing so, they hold the potential to improve health outcomes and reduce the disease burden.

Despite the demonstrated value of integrating genomics data into ML models, the feasibility of genetic testing in routine screening remains a challenge. While conventional biomarkers such as HbA1c and fasting plasma glucose (FPG) remain the gold standard for diagnosis, ML-based models incorporating genomic risk scores may offer additional value by improving risk stratification, particularly in younger individuals who may not yet exhibit abnormal metabolic markers but have a genetic predisposition to T2D. Identifying high-risk individuals early, before the overt appearance of clinical symptoms, could lead to more successful preventive interventions, reducing long-term disease burden.

Although the inclusion of genomic data led to statistically measurable improvements in model performance (e.g., +0.865 % in GBM), the clinical utility of such modest gains warrants careful consideration. In practice, even small improvements may be meaningful at the population level, particularly for early risk stratification or when integrated into multi-factorial decision tools. However, the added cost, logistical demands, and current limited accessibility of genotyping may challenge routine clinical implementation. Thus, while our findings support the potential value of genetic information, especially in younger individuals, its adoption would likely depend on further cost-benefit evaluations and integration into broader risk assessment frameworks.

Although this study demonstrates the feasibility of incorporating genomic data into risk models, prospective research is needed to determine whether early identification improves health outcomes. Future research should also explore whether integrating genetic risk scores enhances risk stratification beyond traditional metabolic markers and assess their cost-effectiveness in real-world screening settings.

From a mathematical perspective, adding genomic data stabilizes prediction. Statistical decision problems are fundamentally inverse problems, which are inherently unstable when affected by slight variations in data [53]. The model stabilizes by incorporating additional information, such as genomic features, reducing uncertainty and enhancing predictive power.

One way to visualize this scenario geometrically is to imagine the minimization of an error function that is not convex and is plagued by local minima, like the valleys and basins in a rugged landscape. Variations in the data and how we measure the loss function being minimized will forcefully lead to changes in the location of the minima, often substantially. However, adding extra information (in our case, using genomic data) helps smooth the landscape and reduce the number of local minima. As a result, the model becomes more robust, benefiting from the enhanced predictive power the genomic information provides.

An interesting analogy can be drawn by looking at computerized tomography, such as X-ray tomography [54]. In this context, we try to reconstruct a density in the interior of a body from finitely many non-invasive external measurements. The interior density, in principle, lives in an infinite-dimensional space, making the reconstruction problem ill-posed. However, the stabilization of the reconstruction comes from introducing *a priori* information, namely, the requirement that the resolution is limited and thus the bandwidth of the interior density must be bounded. Many reconstruction algorithms in computerized tomography exploit this principle, as restricting the bandwidth enhances the robustness of the reconstruction process. Similarly, regularization techniques are employed in mathematical finance to address ill-posed problems, such as the reconstruction of so-called volatility surfaces [55], [56]. In this context, regularization by discretization algorithms has proven effective. Summing up, it can be proposed that, at the mathematical level, incorporating genomic data acts as a form of regularization, stabilizing the inherently ill-posed problem of T2D risk prediction.

Accurately predicting diabetes progression can facilitate early diagnosis and enable healthcare professionals to personalize treatment to individual needs, ultimately reducing healthcare costs and improving patient outcomes [57]. Integrating clinical and genomic data creates a more comprehensive risk profile, essential for developing personalized treatment plans.

## 6 Strengths and limitations

A key strength of this study was the inclusion of clinical and genomic data across two ancestrally distinct populations. Including the UK Biobank dataset (N = 31,620) provided a robust platform to validate the findings and evaluate the predictive performance of the models in a larger population. This strengthens the generalizability of the results to individuals of European ancestry. Although the UK Biobank differs from the Lebanese discovery cohort in ancestry and environmental exposures, this cross-ancestry validation was intentionally performed to evaluate the stability and transferability of our predictive models. Understanding how well models trained in one population perform in another is crucial for real-world implementation of precision medicine tools. In this study, the final set of SNPs was obtained by applying stringent quality control measures and selecting one representative SNP per gene from an initial set of 135. This strategy was designed to reduce redundancy, minimize multicollinearity, and improve model interpretability, particularly within machine learning frameworks. While this gene-level pruning may limit the inclusion of potentially informative variants, it enhances model stability and biological plausibility, supporting better generalizability across datasets. Since the primary aim of this study was to evaluate the predictive value of targeted SNPs previously implicated in T2D, this prioritization was intentional rather than exploratory. We acknowledge, however, that this may favor loci with prior associations. Limitations of the study include the cross-sectional design, which limits causal inference and temporal associations, as the models used classify individuals as having T2D or not at the time of assessment rather than predicting future T2D onset. While classification models are valuable for identifying undiagnosed diabetes cases and individuals at high risk based on current clinical and genetic profiles, longitudinal studies are needed to assess predictive accuracy over time. Future research could integrate machine learning with longitudinal datasets to improve early risk assessment and guide preventive interventions. In addition, although the proportion of excluded individuals due to missing data was relatively small (12–13 %), this step may introduce some degree of selection bias. However, it was necessary to ensure a consistent and complete feature set across both discovery and validation datasets. Future studies could explore the use of data imputation methods or sensitivity analyses to assess the impact of missingness on model performance and generalizability.

Additionally, although clinical measures such as HbA1c and FPG remain the gold standard for T2D diagnosis and screening, this study explored the potential benefit of incorporating genomic data for improved risk stratification. Integrating other omics data, such as metabolomics and proteomics, and investigating underlying pathways could provide deeper insights and support the development of more targeted and personalized interventions for T2D risk prediction. Finally, while this study focuses on using targeted SNPs previously linked to T2D, this approach may not capture the cumulative effects of many variants with smaller individual contributions.

To enhance the accessibility and translational potential of this work, the full R codebase for preprocessing, training, and evaluation is publicly shared on https://github.com/cynthia-hg/T2D-ML-Prediction. The final model was deployed as an interactive Shiny web application on https://huggingface.co/spaces/T2D-Research-Team/T2D_ML. Relevant clinical and genotypic variables can be entered to obtain T2D risk predictions, promoting transparency, reproducibility, and broader adoption of the tool into clinical decision-support systems.

## 7 Conclusion

Machine learning models integrating clinical and genomic data offer a powerful strategy for improving early T2D detection, especially in younger individuals. Age-stratified analysis showed that genomic features benefit younger populations more, enhancing early identification and guiding personalized intervention strategies. As AI evolves, such models may be integrated into clinical workflows and public health initiatives. This can facilitate the proactive management of T2D risk, ultimately improving T2D prevention and management while reducing the global disease burden.

## CRediT authorship contribution statement

**Al Hageh Cynthia:** Writing – review & editing, Writing – original draft, Visualization, Methodology, Formal analysis. **Henschel Andreas:** Validation, Formal analysis. **Zhou Hao:** Methodology, Formal analysis. **Zubelli Jorge:** Validation, Formal analysis. **Nader Moni:** Writing – review & editing. **Chacar Stephanie:** Writing – review & editing. **Lakovidou Nantia:** Formal analysis. **Hatzikirou Haralampos:** Writing – review & editing, Validation, Methodology. **Abchee Antoine:** Writing – review & editing, Data curation. **O’Sullivan Siobhán:** Writing – review & editing. **Zalloua Pierre:** Writing – review & editing, Supervision, Resources, Project administration, Methodology, Investigation, Funding acquisition, Conceptualization.

## Ethics approval and consent to participate

Ethics approval: [IRB#: LAU.SOP.PZ1.2007.R4.6/November/2015]

## Declaration of Competing Interest

The authors declare no competing interests

## Data Availability

The full R code for data preprocessing, model training, and evaluation is publicly available on **GitHub**. Additionally, the final trained model is deployed as a user-friendly Shiny web application on **Hugging Face Spaces** for interactive use. Access to the underlying datasets analyzed during the current study can be provided by the corresponding author upon reasonable request.
